# Size effect of Au/PAMAM contrast agent on CT imaging of reticuloendothelial system and tumor tissue

**DOI:** 10.1186/s11671-016-1650-z

**Published:** 2016-09-26

**Authors:** Wei Wang, Jian Li, Ransheng Liu, Aixu Zhang, Zhiyong Yuan

**Affiliations:** 1 Key Laboratory of Cancer Prevention and Therapy, National Clinical Research Center of Cancer, Department of Radiation Oncology, Tianjin Medical University Cancer Institute and Hospital, Huan-Hu-Xi Road, He Xi District, Tianjin, 300060 China; 2 Department of Radiology, Tianjin Hospital, Tianjin, 300060 China

**Keywords:** Size effect, Computed tomography, Au nanoparticles, Reticuloendothelial system, Tumor tissue, Passive targeting

## Abstract

Polyamidoamine (PAMAM)-entrapped Au nanoparticles were synthesized with distinct sizes to figure out the size effect of Au-based contrast agent on CT imaging of passively targeted tissues. Au/PAMAM nanoparticles were first synthesized with narrow distribution of particles size of 22.2 ± 3.1, 54.2 ± 3.7, and 104.9 ± 4.7 nm in diameters. Size effect leads no significant difference on X-ray attenuation when Au/PAMAM was ≤0.05 mol/L. For CT imaging of a tumor model, small Au/PAMAM were more easily internalized via endocytosis in the liver, leading to more obviously enhanced contrast. Similarly, contrast agents with small sizes were more effective in tumor imaging because of the enhanced permeability and retention effect. Overall, the particle size of Au/PAMAM heavily affected the efficiency of CT enhancement in imaging RES and tumors.

## Background

Being different with classical diagnostic imaging, molecular imaging sets forth to probe the molecular abnormalities that are the basis of disease rather than to image the end effects of these molecular alterations [1, 2]. The physicochemical features of the imaging agent, such as morphology and particle size, play an important role in image quality.

Although CT imaging is not commonly used as a molecular imaging tool in clinical practice because of the fine performance in high spatial resolution and short acquisition time, CT-relevant targeted and specific contrast agents were also rapidly developed recently, aiming to a strongly selective X-ray attenuation and corresponding enhanced contrast. The specifically physical features of light scattering and surface plasmonic properties made Au nanoparticles (AuNPs) promising materials as CT contrast agents for molecular imaging. Up to date, iodine with high X-ray attenuation efficiency is still the most common CT contrast agent. Comparing with iodine, Au has a larger atomic number (79) and electric density (19.32 G/cm^3^), leading to greater coefficient of X-ray attenuation [3, 4]. Previous reports stated that AuNPs serves as the non-cytotoxicity agents, and does not involve in biological process, where no biological toxicity risk such as renal toxicity and vascular permeation will be introduced [5].

As CT contrast agents, dendrimers-entrapped AuNPs were extensively studied, and the previous studies mainly focused on the in vitro efficiency of X-ray attenuation [6, 7] and targeting delivery [8]; however, the size effect on bioapplications were still not totally understood. After being coated with polyethylene glycol (PEG) or dendrimers, blood circulation time was markedly prolonged, and improved biocompatibility was achieved [9–11]. Thus, PEG-coated AuNPs and dendrimers-coated AuNPs were normally regarded as contrast agent of blood pool and lymph node, further applied in tumor-related diagnosis [12, 13]. The flexibility of surface modification made multimodal imaging, such as CT/MR and CT/optical dual modal imaging, achievable according to the conjugation of other imaging tracers [14–16].

Introduction of targeting molecules such as specific protein, biomarkers and aptamer, make the contrast agents actively targeting specific molecules. Although active targeting is the common mechanism of contrast enhancement, the passive targeting is rightfully inevitable, and dominates the distribution of Au/polyamidoamine (PAMAM). Actually, targeting efficiency is partially determined by the morphology and size, so the associated effect is not negligible. Previous studies have shown that morphology may cause significant differences on the biodistribution and have an obvious effect on blood circulation [17, 18]. Correspondingly, distinct size may also bring factual effect both in vitro and in vivo. As nanoscale particles, because of the passively targeting for the reticuloendothelial cells and the enhanced permeability and retention (EPR) effect in the blood vessels of tumor tissue, the accumulation of Au/PAMAM in reticuloendothelial system (RES) macrophages and tumor tissues closely depends on the particle size. Therefore, it is necessary to investigate the size effect on CT imaging of the passively targeted organs and tissues.

So far, Au nanoparticles with uniform size between 5 and 200 nm were widely applied in the field of not only CT imaging but also the drug delivery [19, 20] and radiosensitizer [21]. The aim of this study is to synthesize Au/PAMAM nanoparticles with distinct diameters and explore the influence of diameter on X-ray attenuation in vitro and CT imaging in vivo, particularly on RES uptake and EPR effect, so as to determine the optimal size of Au/PAMAM as contrast agents.

## Methods

### Synthesis of Au/PAMAM

**Fig. 1:**
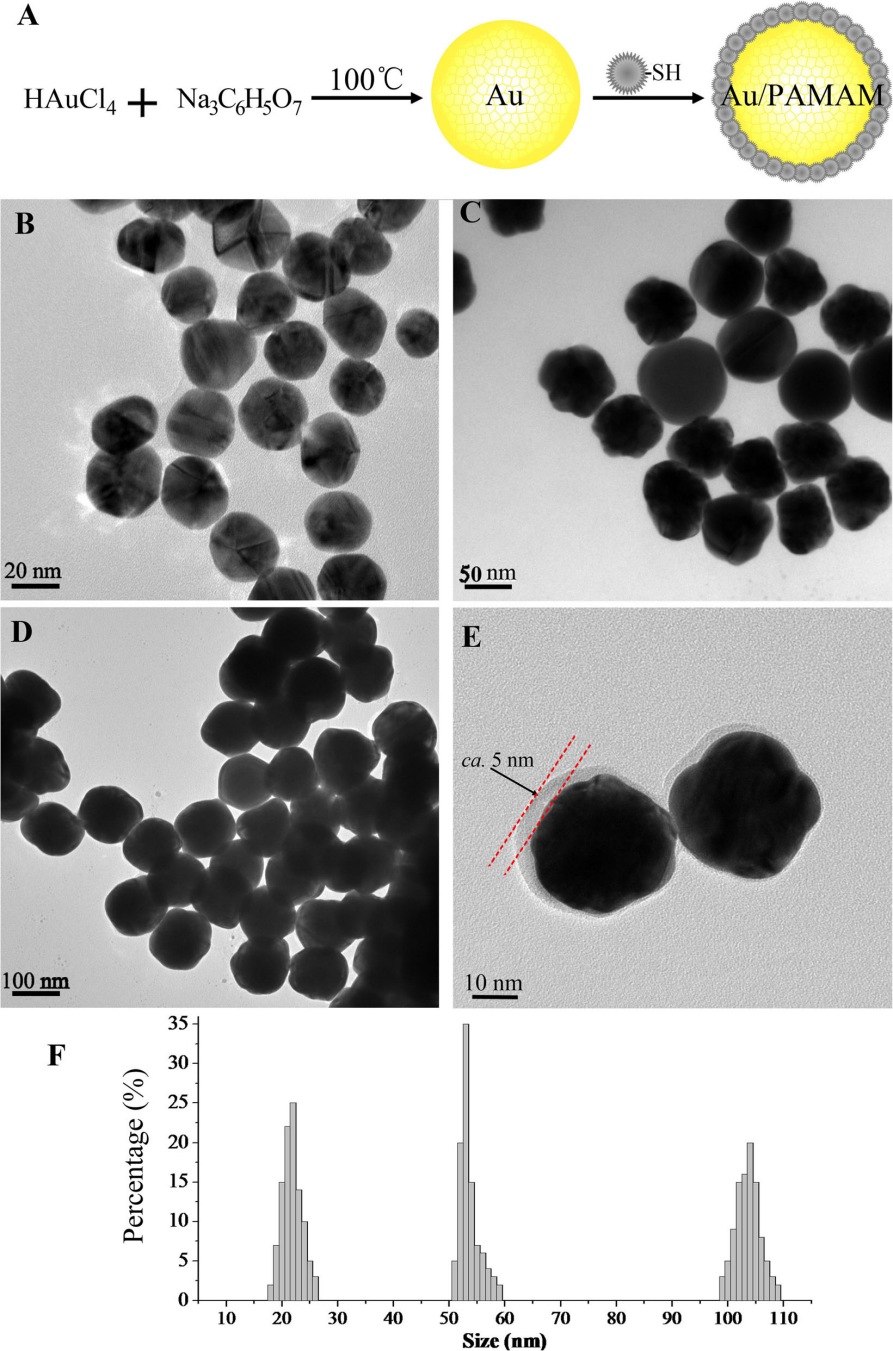
Synthetic scheme and characteristics of Au-based CT contrast agents with gradient sizes. **a** Synthesis of PAMAM-entrapped Au. **b**–**d** TEM of Au nanoparticles in 22.2 ± 3.1, 54.2 ± 3.7, and 104.9 ± 4.7 nm. **e** Typical example of PAMAM-entrapped Au. **f** Size distributions of Au with three distinct sizes

The schematic procedure of Au/PAMAM contrast agents is shown in Fig. 1a. HAuCl_4_ was dissolved in 100 mL distilled water at 0.1 mg/mL and then heated to 100 °C in nitrogen atmosphere. A suitable amount of sodium citrate solution at 10 mg/mL was added as a reducing agent, and reaction system was refluxed for another 10 min. After naturally cooling down, Au nanoparticles were collected and purified via centrifuging at 12,000 rpm for 1 min. Surface coating was performed following an established method [22, 23]. The fifth generation (G5.0) PAMAM dendrimers in methanol were mixed with *N*-hydroxysuccinimidyl 3-mercaptopropanoate in 1:1 mixed solution of tetrahydrofuran and methanol and vortexed for 1 h at room temperature. The PAMAM-SH and AuNPs reacted overnight utilizing the strong affinity between thiol groups and AuNPs and product was collected via centrifuging as well.

### Characterization of particle size and X-ray attenuation

Morphology of Au nanoparticles was observed by transmission electron microscopy (TEM) (H-800; Hitachi, Chiyoda, Tokyo, Japan) operating at 200 kV. A region of interest (ROI) with more than 100 particles was outlined in the TEM images, and particle distribution was analyzed using ImageJ. Au/PAMAM solution was concentrated and quantified with inductively coupled plasma-atomic emission spectrometry (ICP-AES). X-ray attenuations of diluted Au/PAMAM solution were measured at concentration ranging from 0.002 to 0.1 mol/L (0.002, 0.005, 0.01, 0.02, 0.05, 0.1 mol/L) by a CT scanner (Somatom Sensation 16; Siemens Healthcare, Forchheim, Germany) with tube voltage 120 kV, tube current 50 mA, field of view (FOV) 25 cm, matrix 512 × 512, slice thickness 1 mm, and gap 0 mm. Contrast agents with distinct sizes were measured in the same condition and with three replications.

### CT scanning

Nine A549 tumor-bearing BALB/c nude mice (weight 20–25 g, female) were recruited in this research. Animal care and all experimental procedures were approved by and followed the guidance of the Animal Care Committee of Tianjin Medical University Cancer Institute and Hospital. Tumors were located in the right posterior limb, and mice were raised in a SPF-class environments. Au/PAMAM contrast agent was injected intravenously at a dosage of 100 μL (0.1 mol/L, in 0.9 % sodium chloride injection) per 20 g body weight. Mice were scanned with a micro-CT scanner (VECTor^+^/CT; Milabs B.V., Netherlands) at 1 and 4 h post injection under anesthesia with the following parameters: tube voltage 55 kV, tube current 615 μA, FOV 45 mm, slice thickness 10 μm, and gap 0 mm. Quantitative analysis of CT values of ROIs at corresponding time points were performed. Attentions were focused on the size effect of Au/PAMAM on enhancements of image contrast on RES, particularly on the liver, and the size effect of Au/PAMAM on enhancements of image contrast on xenograft tumor.

### In vitro and ex vivo verifications

For Au/PAMAM CT imaging of tumor, contrast agent extravasates through the enlarged endothelial gaps and deposits in the loose connective tissue space. The whole procedure heavily depends on the particle size. Due to low concentration of gold in vivo, after the CT scanning, mice were sacrificed for quantitative analysis of Au element, which was conducted by ICP-AES. The same measurements were performed for the quantitative analysis of Au element in the liver as well. In order to testify the biosafety in acute period, tumorous tissues was stained via HE staining to observe the Au/PAMAM induced changes of cell morphology.

In order to verify the observations on CT imaging of RES-related organs, the size effect on in vitro endocytosis was evaluated on murine macrophage-like RAW264.7 cells; 10^6^ macrophages were incubated with 0.1 μg Au/PAMAM in 0.5 mL DMEM for 1 and 4 h, and cellular uptake efficiencies were quantified by a UV-Vis spectrophotometer (Alpha 1506; LASPEC, Shanghai, China) via the measurements of absorbance of Au/PAMAM at 537 nm before and after the incubation.

## Results

As shown in Fig. 1b–d, f, sizes of Au nanoparticles were restricted as 22.2 ± 3.1, 54.2 ± 3.7, and 104.9 ± 4.7 nm in diameters with narrow and symmetric distributions. Particle sizes were controlled by the amount of reducing agent. During the synthetic procedures, 1.5, 1, and 0.4 mL sodium citrate solution (10 mg/mL) corresponded to AuNPs of 22.2, 54.2, and 104.9 nm, respectively. The less reducing agent produced less crystal nucleus during the early period of synthesis and determined the size of final product. The size of crystal nucleus grown slowly in this gentle reaction condition leading to the narrow distribution of particle sizes. As shown in Fig. 1e, the PAMAM coating covered AuNPs with a single layer of ca. 5 nm. The surface coating utilizes the strong affinity between AuNPs and thiol groups, so the existing PAMAM cover prevented the further coating of PAMAM on the surface, leading to the single layer of surface coating and functionalizing the particle surface with abundant amino groups.

**Fig. 2:**
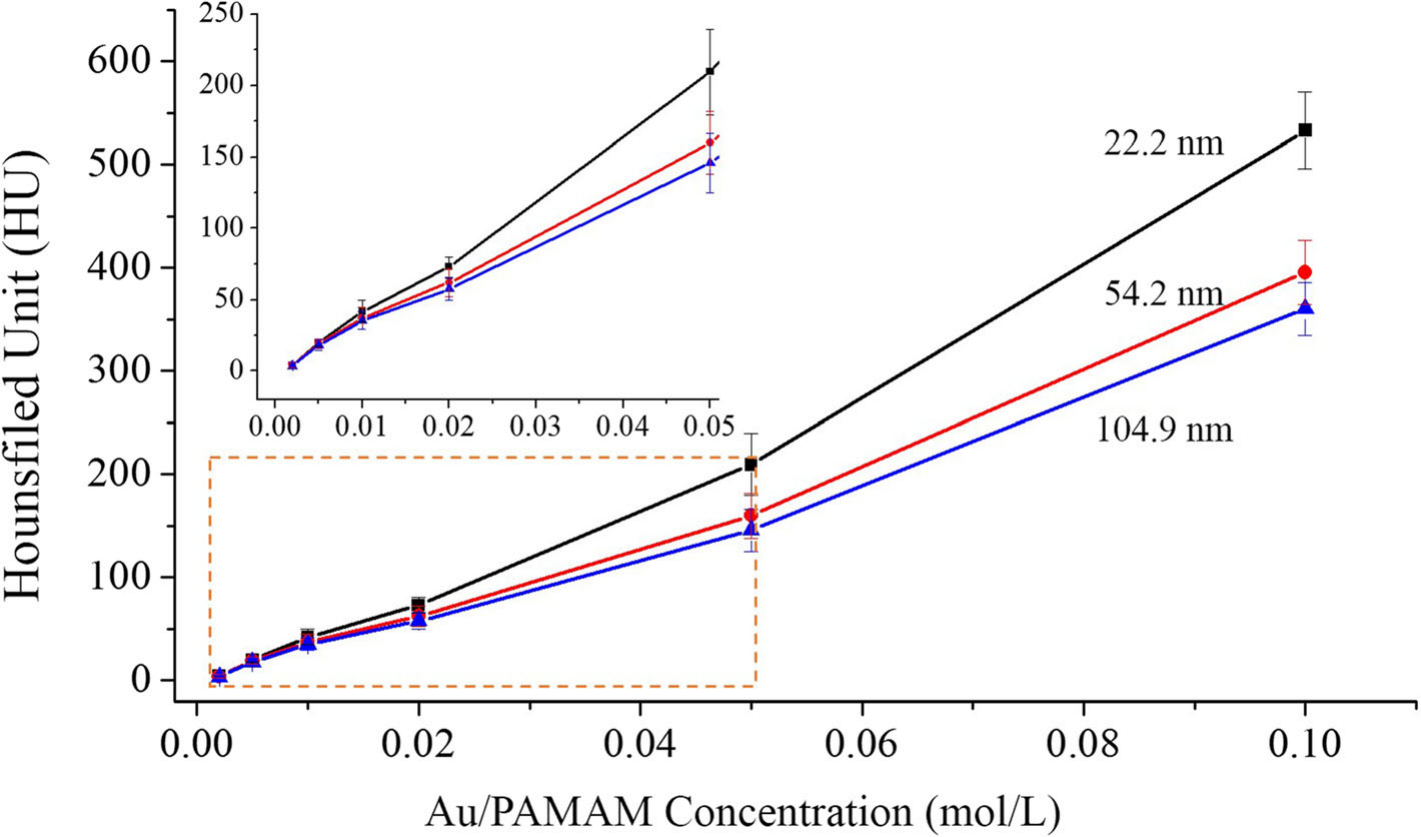
Au/PAMAM lead X-ray attenuation depending on concentration and particle size. The curve of HU values without significant difference were circled in the *orange box* and amplified in the *upper left corner*

As depicted in Fig. 2, Au/PAMAM had a linearly increased enhancing effect on X-ray attenuation relied on the concentration of contrast agents. As shown in the amplified graph, size did not lead to a significant difference on X-ray attenuation for contrast agents with concentration ≤0.05 mol/L.

**Fig. 3:**
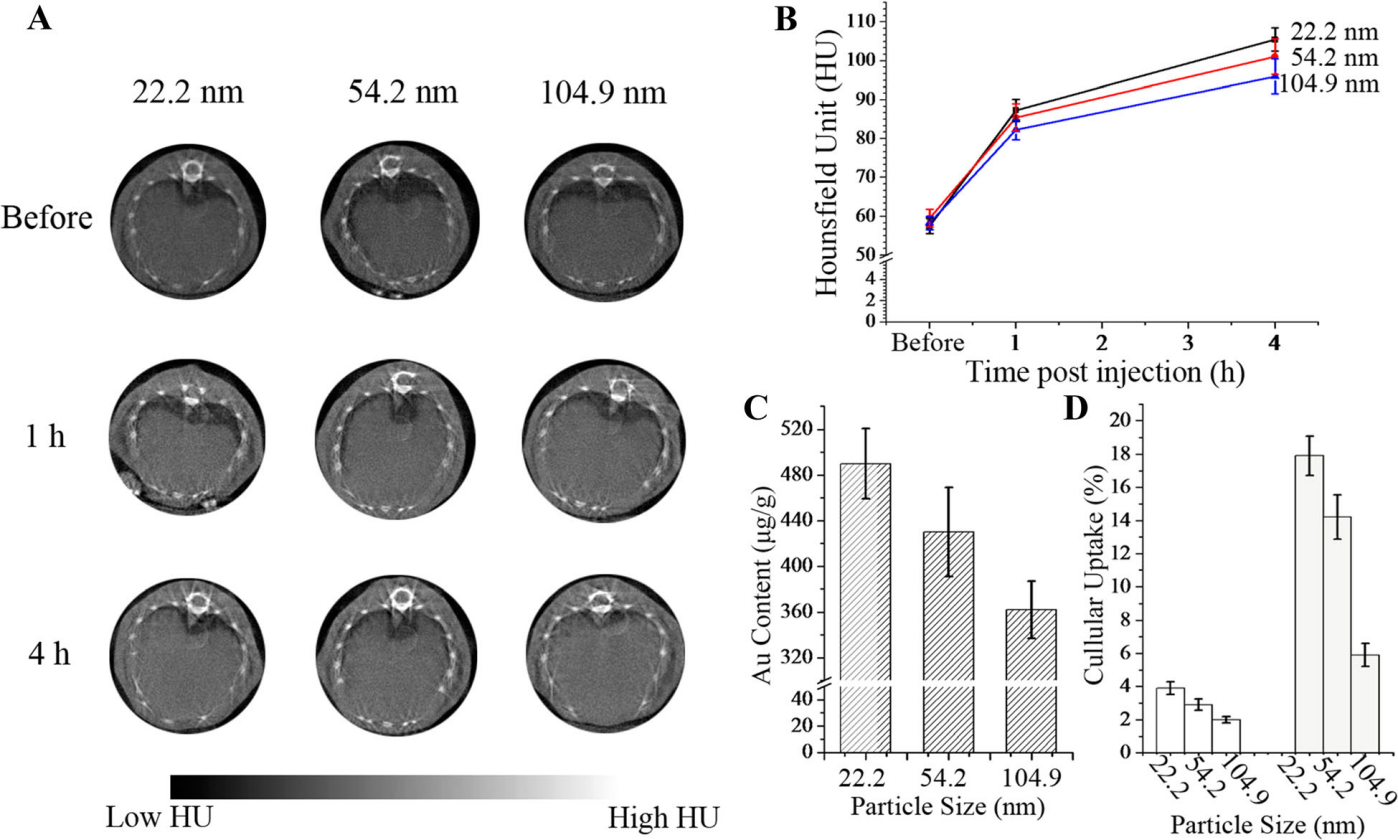
Size effect of Au/PAMAM contrast agent on CT imaging of reticuloendothelial system. **a** Size effect on liver imaging, the typical images of transverse slices of the top of liver were given. **b** Detailed CT values of liver imaging. **c** Au content in corresponding livers detected via ICP-AES at 4 h post injection. **d** In vitro verification of size effect on endocytosis tested after 1 h co-incubation (*blank columns*) and after 4 h co-incubation (*grey columns*) with Au/PAMAM concentration at 10^−7^ μg/cell

The mean CT values of soft tissues were 33.6 Hounsfield unit (HU) (before injection), 36.9 HU (for 22.2 nm), 36.2 HU (for 54.2 nm), and 35.9 HU (for 104.9 nm) at 4 h post injection, and no significant difference was detected. For the enhanced CT imaging of the liver (Fig. 3a), CT values increased along with the accumulation of Au/PAMAM, meanwhile, the increase depended on the particle size (Fig. 3b), where the relatively small Au/PAMAM accumulated in the liver more than the bigger ones significantly (Fig. 3c). Size effect on enhancement of liver imaging was observed that ΔHU = 48.0 for 22.2 nm, ΔHU = 41.6 for 54.2 nm, and ΔHU = 37.3 for 104.9 nm were detected at 4 h post injection. For the in vitro verification of the size effect on endocytosis tested after 1 or 4 h co-incubation, a more obvious size effect-induced difference on cellular uptake was detected that Au/PAMAM with small sizes tended to uptake more than others (Fig. 3d).

**Fig. 4:**
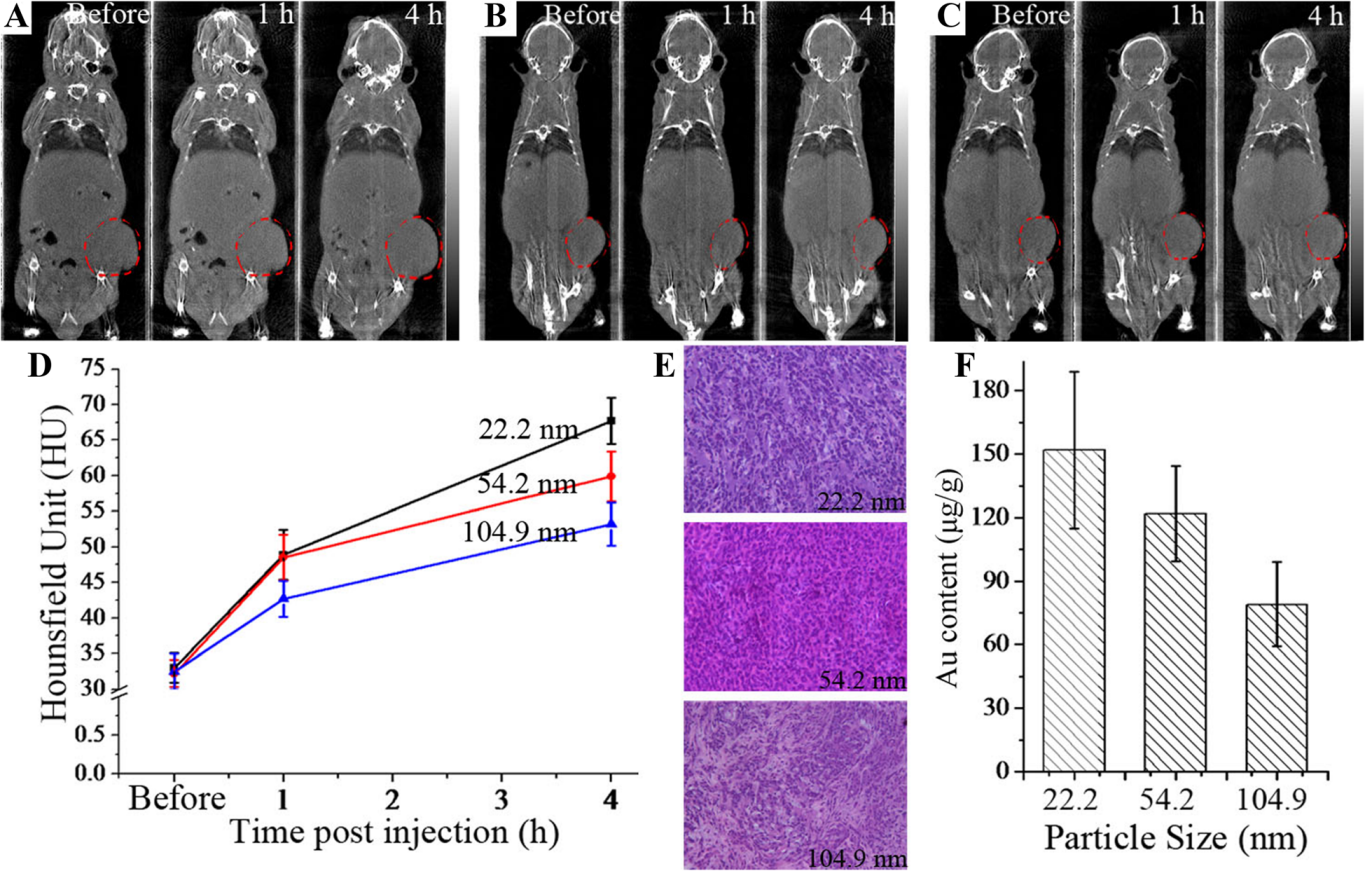
Size effect of Au-based contrast agent on CT imaging of subcutaneous A549 tumor. **a**–**c** CT imaging of tumor with 22.2, 54.2, and 104.9 nm Au/PAMAM injected. **d** Detailed CT values of tumor imaging. **e** HE staining of tumor tissues where no changes of cell morphology were observed. **f** Au content in corresponding tumors detected via ICP-AES at 4 h post injection

For the enhanced CT imaging of tumors (Fig. 4a–c), tumorous tissues were more clearly outlined. In the quantitative analysis (Fig. 4d), CT values increased along with the accumulation of contrast agents, and size effect-induced differences on ΔHU were amplified due to the lasting accumulation of Au/PAMAM. No Au/PAMAM-induced changes of cell morphology was observed on HE staining (Fig. 4e), proving the biosafety in acute period after injection. As the fundamental reason of difference on CT enhancements, Au content in tumor tissues heavily relied on the particle size (Fig. 4f), but an obviously lower level of Au accumulation in the tumor was observed when compared with Au content in the liver, this is why the ΔHU for tumor imaging was smaller than the ΔHU in liver imaging.

## Discussion

Two methods were commonly used in synthesis of Au/PAMAM. For the Au/PAMAM with relatively small size, PAMAM was utilized as the core, and Au element was filled in the cage of PAMAM or attached to the surface of PAMAM [9, 10]. Due to the limitation of PAMAM generation, the size of this kind of Au/PAMAM was restricted to less than 15 nm. For Au/PAMAM with bigger size, AuNPs were used as the core, and thiolated PAMAM was covered to the surface just like the method used in this research. PAMAM with G4.0 and G5.0 was easily being attached to the surface to form the stable complexes, but the coating of PAMAM with lower generation was less stable because of the easy aggregation resulted from the low degree of grafting and related flexibility. In the design of Au-based contrast agents, PAMAM stabilized the nanoparticles, improved the solubility and biocompatibility, and further functionalized the AuNPs with more potentials, such as active targeting and multimodal imaging modalities. The targeting delivery to specific target or controlled release in targeted tissues can be realized through surface functionalization with targeting molecule. For example, the dual-targeting for prostatic cancer was realized via the conjugation of two targeting molecules on PAMAM [24]. Other imaging tracers can be labeled to Au/PAMAM via chemical reaction with amino groups or via chemisorption. The labeling of another imaging tracers, such as fluorescent dyes and radionuclide, can realize the dual modal imaging with enhanced CT [15, 16]. Furthermore, diagnosis and therapy can be combined in one agent, and imaging with more than two modalities can be achieved based on the functionalization of Au/PAMAM as well.

Size heavily influenced on the in vivo endocytosis, which is dominated by macrophages, but endocytosis will also be affected by the intensity of blood flow and elimination rate. As shown in the in vitro test, and proved in previous research, the rate of particle uptake depends strongly on the NP size, with the smaller sizes internalized much more quickly than the larger ones [25]. Relatively small particle size corresponds to heavy endocytosis, forming the main reason of high uptake in the liver, which contained abundant macrophages. Hence, the primary cause leading to the difference on CT imaging of RES is not the size effect on X-ray attenuation, which is proved not obvious when concentration was ≤0.05 mol/L, but the size effect-induced difference on endocytosis efficiency of macrophages. However, due to the confounding factors, such as blood and vessels penalty, the size effect-induced in vivo difference on CT values was not as obvious as the size effect observed on endocytosis in vitro. During the early period (1 h post injection), CT contrast was already enhanced, but the size effect-induced difference on CT imaging of the liver was not totally exhibited. The enhancements on CT contrast were gradually strengthened along with the increase of particle deposition. Meanwhile, the difference among the three kinds of Au/PAMAM on CT contrast also increased along with the gradual increase of particle deposition. For most contrast agents, imaging was performed soon after the injection. The longer time means more confounding factors for the imaging results, such as the individual difference. Secondly, based on another research in our lab [21], most AuNPs will be expelled at 6 h post injection or more. So, as a potential preclinical research, imaging research focus on the 1 and 4 h post injection, and the difference resulted from longer time was not explored.

For the EPR effect involved in tumor imaging, although nearly all the particles with size less than 700 nm can passively target tumor, it is obvious that the size effect exists for the penetration through new vessels [26]. The smaller size facilitated the Au/PAMAM in extravasating through the enlarged endothelial gaps in tumors, leading to a more obviously enhanced CT contrast. Utilizing this phenomenon, the drug carrier in smaller size is normally with an increased delivering efficiency [18].

This research was referable to passive targeting of other nanoparticles. The size effect of Au/PAMAM on passive targeting helped to figure out the in vivo mechanism of AuNPs and similar nanoparticle-based agents. Both optimizing the imaging of passively targeted organs and providing reference for the design of actively targeting imaging agents were achieved. Based on the observation on Au/PAMAM-based CT imaging of the liver and tumor, among the tested size range, 22.2 nm was the best choice in drug designation. The superiority of enhanced passive targeting resulted from small size, plus the active targeting ability of targeting molecules, will doubly promote the tumor-targeted imaging. However, the passive targeting for RES-related organs will be strengthened at the same time. In active targeting delivery which aims to avoid RES uptake, the slight increase of the particle size will decrease the passive target to RES effectively, but will not heavily influence on the extravasating through the enlarged endothelial gaps in tumors. Additionally, the potential defect of longtime retention in macrophages that resulted from small size should be paid more attention when applied in vivo. Actually, 100–200 nm in diameter is the usual size for the nanoparticle-based drugs approved by FDA because of the relatively low RES-related uptake and low cytotoxicity [27]. Hence, a comprehensive consideration on the particle size was need when designing a nanoparticle-based drug.

Due to the low resolution of micro-CT, the contrast resulted from the introduction of imaging agents was not totally exhibited, but proved by the quantitative analysis of CT values. The size effect of contrast agents could be more clearly exhibited in the bigger animal models and with improved scanning techniques.

## Conclusions

The size effect of Au/PAMAM has no obvious influence on X-ray attenuation when Au/PAMAM was ≤0.05 mol/L but does have influence on the CT imaging of living body. The size effect of Au/PAMAM on endocytosis and EPR effect should be paid attention when applied in vivo, where nanoparticles with relatively small size was more efficient in passive targeting and enhancing image contrast.
